# Characterization of XPR1/SLC53A1 variants located outside of the SPX domain in patients with primary familial brain calcification

**DOI:** 10.1038/s41598-019-43255-x

**Published:** 2019-05-01

**Authors:** Uriel López-Sánchez, Gaël Nicolas, Anne-Claire Richard, David Maltête, Mahmoud Charif, Xavier Ayrignac, Cyril Goizet, Jawida Touhami, Gilles Labesse, Jean-Luc Battini, Marc Sitbon

**Affiliations:** 1 Institut de Génétique Moléculaire de Montpellier, University of Montpellier, CNRS, Montpellier, France; 2 Normandie Univ, UNIROUEN, Inserm U1245, Rouen University Hospital, Department of Genetics and CNR-MAJ, Normandy Center for Genomic and Personalized Medicine, Rouen, France; 3 Department of Neurology, Rouen University Hospital and University of Rouen, Rouen, France; 4 INSERM U1239, Laboratory of Neuronal and Neuroendocrine Differentiation and Communication, Mont-Saint-Aignan, France; 5 Department of Neurology, Montpellier University Hospital, Montpellier, France; 6 INSERM U1211, Univ Bordeaux, Laboratoire Maladies Rares, Génétique et Métabolisme; CHU Bordeaux, Service de Génétique Médicale, Bordeaux, France; 7 Centre de Biochimie Structurale, University of Montpellier, CNRS, Montpellier, France; 8 Institut de Recherche en lnfectiologie de Montpellier, University of Montpellier, CNRS, Montpellier, France

**Keywords:** Carrier proteins, Mechanisms of disease, Medical genetics, Neurophysiology, Disease genetics

## Abstract

Primary familial brain calcification (PFBC) is a rare neurological disease characterized by deposits of calcium phosphate in the basal ganglia and other regions of the brain. Pathogenic variants in the *XPR1/SLC53A1* gene, which encodes the only known inorganic phosphate exporter, cause an autosomal dominant form of PFBC. These variants are typically located in the SPX N-terminal domain of the protein. Here, we characterize three XPR1 variants outside of SPX in three PFBC patients with an apparently sporadic presentation: c.1375C > T p.(R459C), c.1855A > G p.(N619D) and c.1886T > G p.(I629S), with the latter identified as the first *XPR1/SLC53A1 de novo* mutation to occur in a PFBC proband. When tested in an *in vitro* physiological complementation assay, the three XPR1 variants were impaired in phosphate export function, although they were normally expressed at the cell surface and could serve as functional receptors for retrovirus entry. Moreover, peripheral blood cells from the p.N619D patient could be assayed *ex vivo* and displayed significantly impaired phosphate export. Our results establish for the first time the clinical and molecular characteristics of XPR1 variants located outside the SPX domain and assert a direct link between these variants, deficient phosphate export, and PFBC. Moreover, we unveiled new structural features in XPR1 C-terminal domain that play a role in phosphate export and disease.

## Introduction

Primary familial brain calcification (PFBC) is a rare neurological disease characterized by the presence of calcium phosphate deposits in the microvessels of the basal ganglia and other brain regions. Clinical signs may start at any age^1^ and comprise mostly movement disorders, cognitive impairment and psychiatric symptoms, and other neurological symptoms^2^. However, up to 42% of the patients can be asymptomatic^1^. PFBC is typically inherited as an autosomal dominant trait with four causal genes identified: *PiT2/SLC20A2*^3^, *PDGFRB*^4^, *PDGFB*^5^ and *XPR1*^6^, which has recently been assigned to the solute carrier (SLC) family of transporters as *SLC53A1* (https://www.genenames.org/cgi-bin/genefamilies/set/752/). Most recently, *MYORG* bi-allelic mutations have also been identified as causing autosomal recessive PFBC^7^.

Interestingly, both *PiT2*/*SLC20A2*^8,9^ and *XPR1/SLC53A1* encode phosphate transporters, with the latter encoding for XPR1, the sole known inorganic phosphate exporter in humans and other metazoans^10^. Five *XPR1/SLC53A1* missense variants have been identified to be causal for PFBC in seven families^6,11^ and functional analyses have shown that the five XPR1 corresponding proteins have reduced cell surface expression and/or were significantly impaired in phosphate export^6,11^. Interestingly, all five damaging variants identified and functionally characterized so far are located in the N-terminal cytoplasmic region of XPR1, with four located within the SPX domain^6,11^. SPX, which is highly conserved among eukaryote XPR1, has been shown to regulate phosphate intracellular levels in yeast and plants^12^. Here, we characterized three novel *XPR1/SLC53A1* missense variants that are located outside of the N-terminal SPX-encoding region, of which two were located in the XPR1 cytoplasmic C-terminal encoding region. We show that the c.1855A > G p.(N619D) C-terminal variant, which has been previously reported as of unknown significance^13^, can now be considered as causal for PFBC and used in a clinical setting. Moreover, DNA sequencing of both parents of the proband allowed us to confirm parenthood and identify the c.1886T > G p.(I629S) C-terminal variant, reported here for the first time, as the first PFBC mutation documented to occur *de novo* in *XPR1/SLC53A1*. Furthermore, we assessed the functional impact of the corresponding mutations on cell surface expression, retroviral receptor function and phosphate export, unveiling for the first time a key and specific role of domains outside the N-terminal regions and the SPX domain in XPR1 phosphate export functions, and PFBC.

## Results

### *XPR1/SLC53A1* variants outside of the region encoding the N-terminal cytoplasmic domain

**Figure 1:**
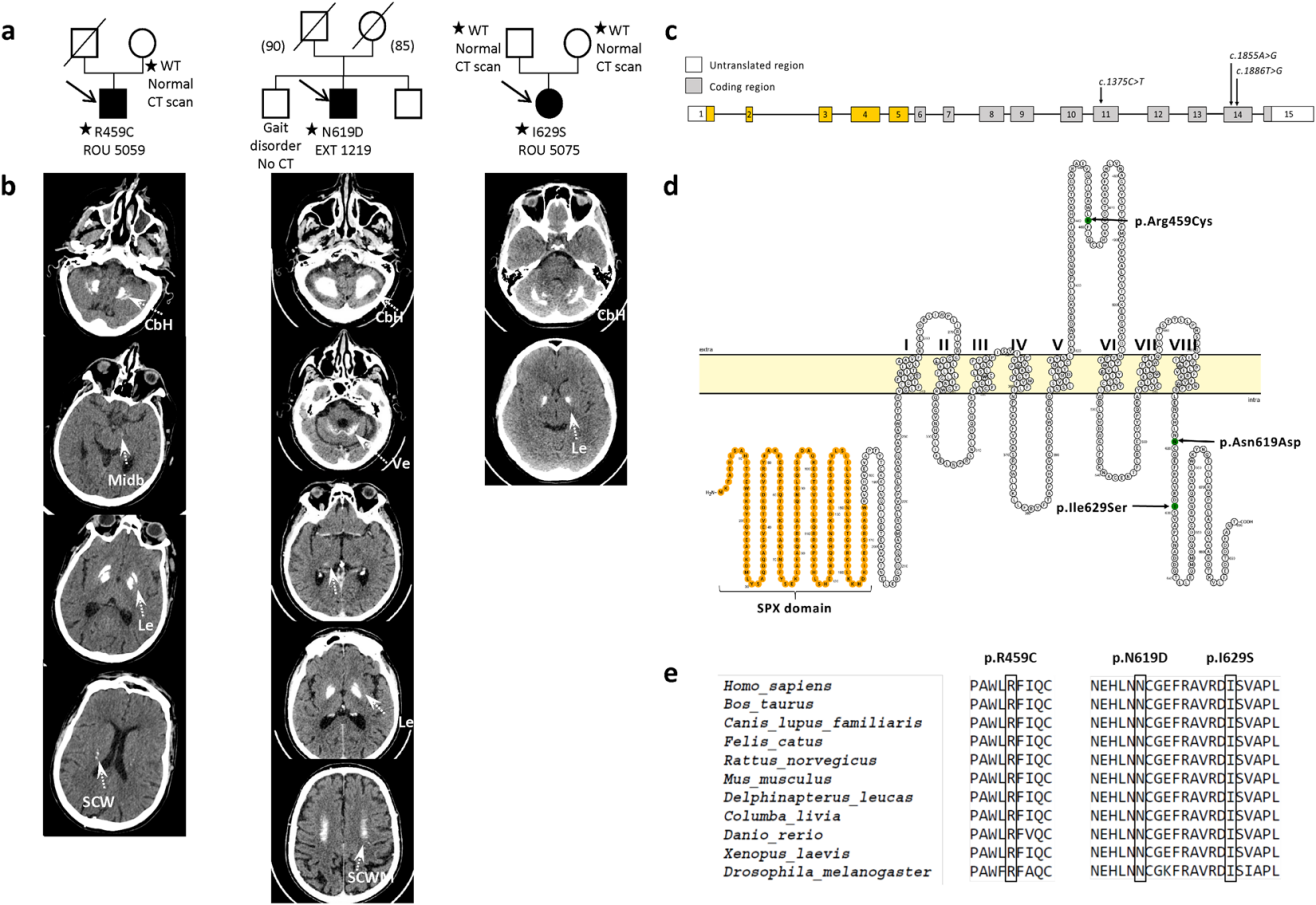
Pedigrees and CT-scans of patients harboring *XPR1/SLC53A1* variants located outside of the SPX-encoding domain. (**a**) Reduced pedigrees of the probands harboring *XPR1/SLC53A1* variants. Open symbol: unaffected; filled symbol: affected (presence of brain calcifications); symbol with a diagonal line: deceased; arrow: proband; numbers in parentheses: age at death; Star: DNA available; WT: wild type. (**b**) CT scans of the probands in axial sections, arrows indicate the different areas of calcifications; Le: Lenticular; T: Thalamic; SCWM: Subcortical white matter; CbH: Cerebellar hemisphere; Ve: Vermis; Midb: Midbrain. (**c**) Schematic representation of the *XPR1/SLC53A1* gene with the SPX-encoding exons indicated in orange, with the changes corresponding to the three PFBC variants indicated on exons 11 and 14 (arrows). (**d**) Schematic representation of the XPR1 protein structure with the SPX domain highlighted in orange, and the three residues corresponding to the two XPR1/SLC53A1 variants p.(R459C and pN619D) and the de novo mutant (pI629S) shown in green. The topological structure was generated using Protter program^39^. (**e**) Alignment of XPR1 orthologs, exclusively from metazoan species, showing the evolutionary conservation of the regions wherein PFBC variants were located.

We report here the phenotypes associated with three *XPR1/SLC53A1* variants. Two were recorded by the French PFBC study group: c.1375C > T p.(R459C) and c.1855A > G p.(N619D); and the third one is a novel c.1886T > G p.(I629S) variant (Fig. 1a,b). The two former variants were mentioned in a recent international study aiming at reporting diagnostic yields^13^, while the latter, identified after the data freeze of the aforementioned study, is entirely novel.

All three variants were heterozygous (Fig. S1). The p.R459C variant maps to exon 11 (NG_050964, NM_004736.3), corresponding to the predicted largest extracellular loop of XPR1/SLC53A1, while variants p.N619D and p.I629S, encoded by exon 14, are both located in the XPR1/SLC53A1 cytoplasmic C-terminal domain (Fig. 1c,d). No particular function for XPR1/SLC53A1 C-term has been described yet. Remarkably, all three variants are located in highly evolutionary conserved regions of XPR1 (Fig. 1e), and are predicted damaging by the MutationTaster^14^, PolyPhen-2^15^, and SIFT^16^ bioinformatics tools. All three variants are absent from control databases, including the genome aggregation database (gnomAD) that gathers over 130,000 individuals (http://gnomad.broadinstitute.org/)^17^.

### Phenotype of the patients harboring the new *XPR1/SLC53A1* variants

#### Patient ROU 5059, c.1375C > T p.(R459C) variant

The patient presented at age 58 years with parkinsonism that started at age 55 by a left upper limb tremor. He was born preterm and presented learning difficulties requiring education in a school for special needs. A diagnosis of mild intellectual disability was performed. In addition, he was treated for high blood pressure, presented a history of arthrosis from age 15 and experienced idiopathic peripheral facial palsy with partial spontaneous recovery at age 58. Upon examination at age 59, he presented bilateral akinetic hypertonic syndrome with left predominance associated to a resting tremor. This parkinsonism was considered as L-Dopa-responsive.

His computed tomography (CT) scan revealed severe bilateral lenticular calcifications as well as moderate bilateral cerebellar hemisphere, bilateral faint supratentorial white matter and faint left midbrain calcifications, with a total calcification score (TCS) of 20/80 (Fig. 1). Brain scintigraphy with ^123^I-Ioflupane showed marked bilateral dopaminergic neuron loss with right predominance. The patient had a positive family history of Parkinson disease for the maternal grandfather (no CT scan), while his mother was asymptomatic and presented a normal CT scan. His father died at the age of 67, with no history of neuropsychiatric disease.

We identified a c.1375C > T p.(R459C) variant in the proband, which was absent in his mother’s DNA. This variant was classified as likely pathogenic (class 4) following the ACMG-AMP recommendations^13^.

#### Patient EXT 1219, c.1855A > G p.(N619D) variant

The patient presented with sudden deafness of unknown cause and unstable gait at the age of 69. He had a negative personal medical history. Upon examination, he presented a mild static cerebellar syndrome. The patient’s CT scan revealed bilateral severe lenticular calcifications as well as bilateral moderate supratentorial white matter, bilateral faint calcification of the thalamus, bilateral severe cerebellar calcification, and faint vermian calcification (TCS = 29/80).

The patient’s 71-year-old brother was reported to present a gait disorder since he was 50. However, neither CT scan nor DNA samples were available for the brother. The father died at the age of 50 after committing suicide.

We identified a c.1855A > G p.(N619D) *XPR1/SLC53A1* variant in this patient. This variant has been initially recorded as of unknown significance (class 3), following the ACMG-AMP recommendations^13^.

#### Patient ROU 5075, c.1886T > G p.(I629S) variant

The patient was 42 years of age when she presented with fluctuant paresthesia of the left upper limb. Her medical history was marked by a diagnosis of celiac disease. She also had a history of migraine without aura. She presented several episodes of paresthesia, sometimes associated with light paresis of the limb or ipsilateral hemiparesis that were not related to her headache episodes. She also complained of overall fatigability and anxiety. Upon examination at age 45, she still presented subjective hypoesthesia of the left hemibody together with left motor fatigability. However, no objective sign was observed, including the absence of pyramidal, extrapyramidal, or cerebellar sign. Brain CT scan of the patient revealed the presence of bilateral moderate lenticular and cerebellar hemisphere calcifications (TCS = 12/80) (Fig. 1).

The patient’s family history was negative. Both parents presented a normal neurological examination with normal CT scans. She presented though a heterozygous c.1886T > G p.(I629S) variant that we could demonstrate to have occurred *de novo*, as neither parent harbored this variant (Fig. S1). We confirmed parenthood using informative microsatellites. Hence, the p.I629S variant could be classified as likely pathogenic (class 4), following ACMG-AMP recommendations.

### All XPR1/SLC53A1 PFBC variants are functionally expressed at the cell surface

To evaluate the impact of *XPR1/SLC53A1* variants on XPR1 expression, we cloned and assayed *WT XPR1*, as well as mutants encoding the p.R459C, p.N619D or p.I629S variants, or the artificial XPR1 deletion construct, p.(L612_T696del), in which the C-terminal cytoplasmic domain was entirely deleted. All constructs were cloned in the pCHIX expression vector. Expression, presence at the cell surface, and retroviral receptor and transport functions were assessed for the PFBC variants, in comparison with those of the WT XPR1 and the p.(L612_T696del) construct.

**Figure 2:**
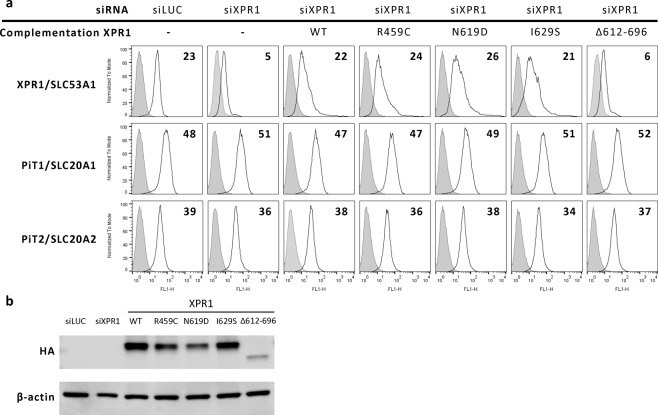
PFBC *XPR1/SLC53A1* variants and XPR1, PiT1 and PiT2 cell surface expression. (**a**) Cell surface detection of XPR1/SLC53A1, PiT1/SLC20A1 and PiT2/SLC20A2 with RBD specific ligands derived from mouse xenotropic MLV, koala endogenous retrovirus, and mouse amphotropic-MLV, respectively (open histograms); HEK293T positive and negative control cells for XPR1/SLC53A1 expression were transfected with either an anti-*luciferase* siRNA (siLUC) or an anti-*XPR1/SLC53A1* siRNA (siXPR1), respectively. WT XPR1/SLC53A1 and variants were assayed for cell surface expression upon co-transfection of siXPR1 alone (−) or in combination with an expression vector coding either for the HA-tagged WT XPR1 (WT), or the PFBC XPR1 C-terminal variants (R459C, N619D, or I629S), or the p.(L612_696Tdel) artificial XPR1 mutant (Δ612–696). Numbers indicate the delta mean fluorescence intensity of a representative experiment (n = 3) compared to the non-specific staining obtained with the secondary IgG antibody (grey histograms). (**b**) Representative immunoblot of HA-tagged XPR1/SLC53A1-containing cell lysates with an anti-HA antibody (upper panel), or with an anti β-actin antibody used as loading control (lower panel).

WT and all three PFBC variants of XPR1/SLC53A1 were detected at the cell surface by flow cytometry using the XRBD ligand, an XPR1-specific ligand previously described^10^. In contrast, the p.(L612_T696del) construct was not detectable at the cell surface (Fig. 2a) despite efficient expression. While all naturally occurring variants of XPR1/SLC53A1 were equivalently and efficiently expressed at the cell surface, they appeared to accumulate at different intracellular levels (Fig. 2b). Also, expression of the PFBC variants had no detectable effect on the cell surface expression of the phosphate importers PiT1/SLC20A1 and PiT2/SLC20A2 (Fig. 2a).

**Figure 3:**
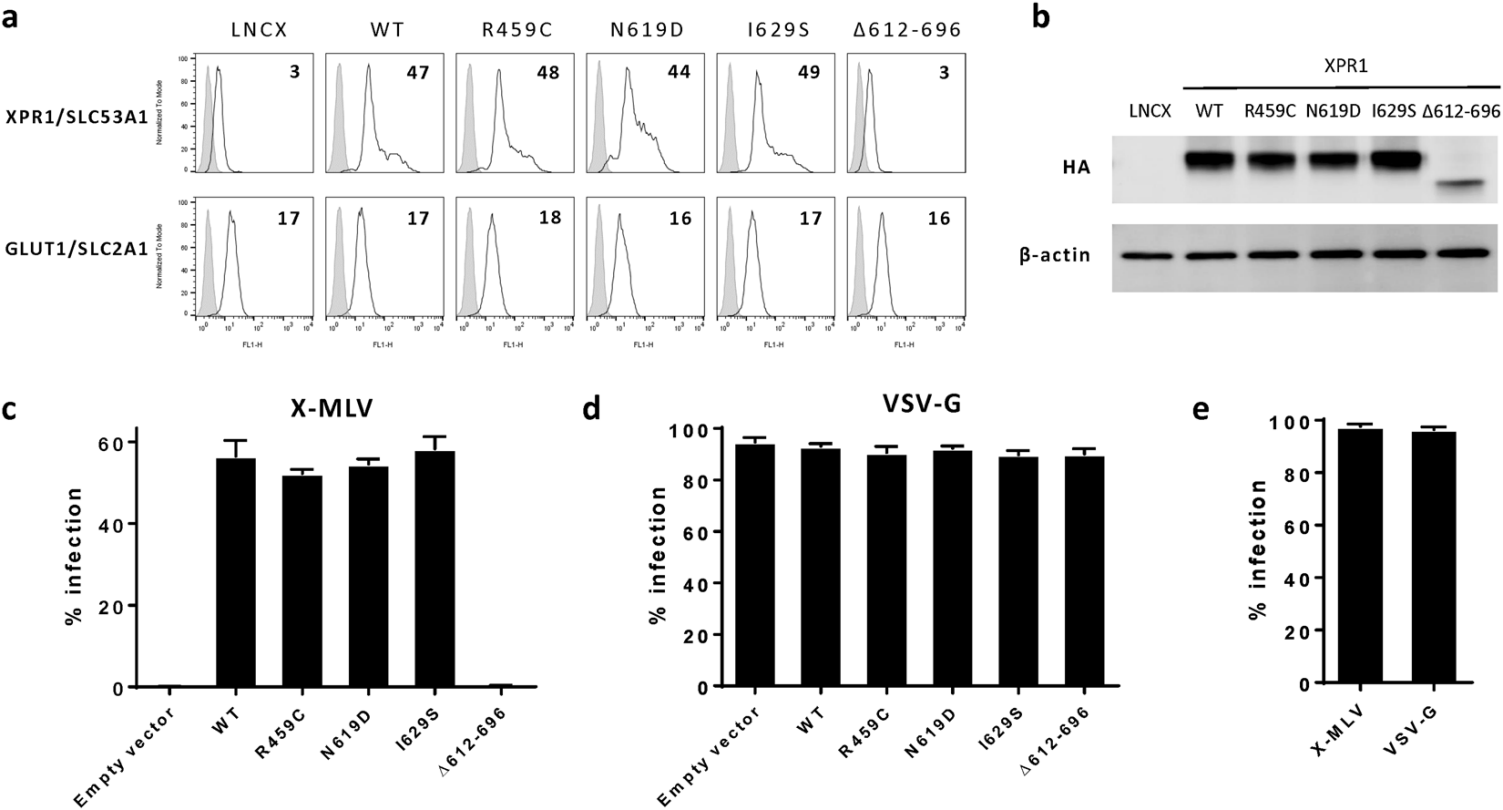
PFBC XPR1/SLC53A1 variants do not alter virus entry. (**a**) Cell surface detection of XPR1/SLC53A1 (upper panel) and GLUT1/SLC2A1 (lower panel) in CHO hamster cell populations stably expressing the control parental expression vector lacking *XPR1/SLC53A1* (LNCX), or encoding either WT XPR1/SLC53A1, or variants p.R459C, p.N619D or p.I629S, or the p.(L612_T696del) deletion mutant. Cells were assayed for XPR1/SLC53A1 and GLUT1/SLC2A1 surface expression with their corresponding XRBD and H2RBD ligands, respectively. Numbers indicate the specific delta mean fluorescence intensity of a representative experiment (n = 3), as compared to non-specific staining with the secondary IgG (grey histograms). (**b**) Representative immunoblot of HA-tagged XPR1/SLC53A1-containing cell lysates from (**a**) with an anti-HA antibody (upper panel), or with an anti β-actin antibody used as loading control (lower panel). (**c**) Analysis of viral infection in CHO cells stably expressing either of WT or XPR1/SLC53A1 variants. Cells were assayed for infection with retroviral vectors pseudotyped with X-MLV Env or (**d**) VSV-G. Two days after infection, cells were analyzed by flow cytometry. Graphics are average values from 3 different wells in one representative experiment (n = 3). (**e**) Infectivity of viral supernatants on control HEK293T susceptible cells.

XPR1/SLC53A1 is the entry receptor of mouse retroviruses, in particular the xenotropic murine leukemia viruses (X-MLV)^18–20^. We therefore monitored PFBC XPR1/SLC53A1 variants for their capacity to serve as cell surface receptor for X-MLV entry and infection. For this purpose, we generated CHO hamster cells, which are refractory to X-MLV entry, as stably expressing either WT human XPR1 or the different PFBC variants. Similar cell surface expression of the WT XPR1 and the three PFBC variants was also observed on hamster CHO cells, while the p.(L612_T696del) construct was not detected at the cell surface (Fig. 3a, upper panel). Of note, the p.(L612_696Tdel) construct reached a significantly lower overall cellular expression in CHO cells when compared to WT and the three PFBC variants (Fig. 3b). All cell lines showed equivalent levels of expression of the glucose transporter GLUT1/SLC2A1 at the cell surface, demonstrating no generic defects or differences in SLC cell surface expression among the different CHO cell lines (Fig. 3a, lower panel). When challenged for retrovirus infection mediated by the X-MLV envelope glycoprotein (Env), we observed that PFBC variants served as cell surface retroviral receptor as efficiently as WT XPR1 (Fig. 3c), rendering CHO cells as efficiently infectable as H293T susceptible cells (Fig. 3e). As expected, cells expressing the p.(L612_696Tdel) XPR1 construct that was not expressed at the cell surface, remained uninfected (Fig. 3c). All CHO cell lines, including the latter, remained equally permissive to retroviral infection mediated by the VSV-G Env which enter cells through a different receptor, indicating that all cell lines had similar abilities to support general retroviral infection past viral entry (Fig. 3d), and that defects in infection by the p.(L612_696Tdel) construct was solely due to the absence of this artificial mutant at the cell surface.

### Deficiency in phosphate export function of the three novel XPR1/SLC53A1 PFBC variants

**Figure 4:**
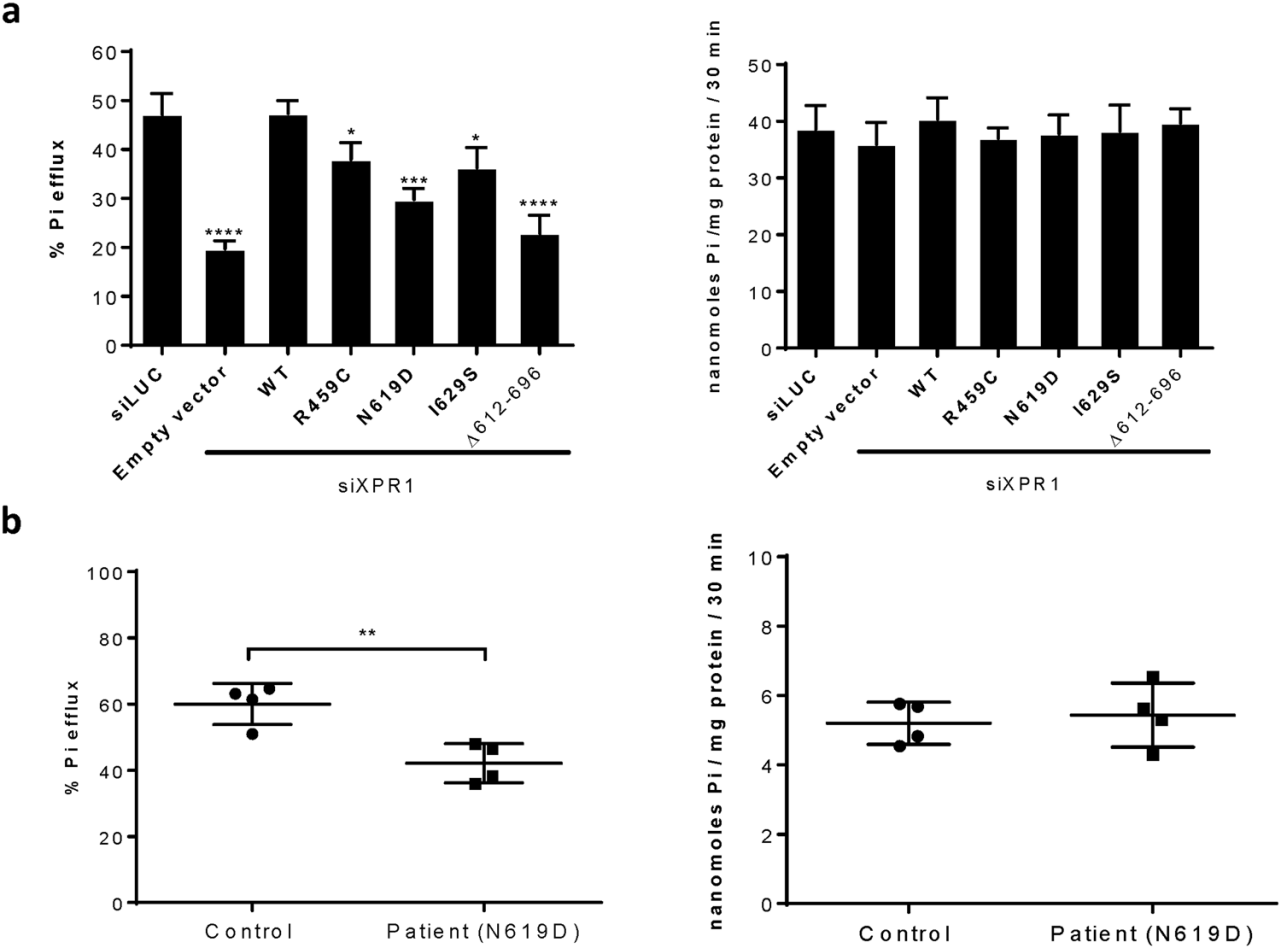
Effect of PFBC mutations on XPR1/SLC53A1-mediated phosphate efflux. (**a**) Efflux and uptake of [^33^P]-phosphate in HEK293T cells transfected with siRNA directed against *luciferase* (siLUC), or *XPR1/SLC53A1* (siXPR1), or with the combination of siXPR1 and an expression vector coding either for the HA-tagged WT XPR1/SLC53A1 (WT), or the R459C, N619D, or I629S PFBC variants, or the artificial XPR1/SLC53A1 mutant Δ612–696. Results are shown as means ± s.e.m. in a representative experiment (n = 3); *P ≤ 0.05; ***P ≤ 0.001; ****P ≤ 0.0001, in comparison to the control (siLUC). (**b**) Efflux and uptake of [^33^P]phosphate in PBMC isolated either from a healthy donor (circles) or from the PFBC patient harboring the p.N619D variant (squares). Efflux is measured as % of released to total cellular [^33^P] phosphate; and uptake is expressed as nmol/mg of protein in 30 min. Bars represent means ± s.e.m **P ≤ 0.01.

XPR1 is a conserved and ubiquitously expressed SLC, which we previously identified as the only known metazoan inorganic phosphate exporter^10^. Phosphate export mediated by XPR1 can be efficiently assessed by a complementation assay, in which the knockdown of endogenous XPR1 by an siRNA directed against *XPR1* 3′UTR and the ensuing decreased phosphate efflux are compensated by the *de novo* transfection of an XPR1 expression vector that is not recognized by the siRNA^10^. Upon introduction of WT XPR1, siRNA-mediated decreased phosphate efflux was fully complemented, while complementation by the p.(L612_696Tdel) construct, which was not expressed at the cell surface, was similar to that of an empty control vector (Fig. 4a, left panel). Noticeably, the three PFBC variants had different levels of complementation for the phosphate exporter function, with the p.N619D variant showing the most marked defect (Fig. 4a, left panel). Importantly, this defect was observed only for phosphate efflux, as phosphate uptake remained unchanged for all constructions, thus confirming the specificity of XPR1 for phosphate export (Fig. 4a right panel).

We had access to peripheral blood mononuclear cells (PBMC) from the patient harboring the p.N619D variant. When assayed *ex vivo* for phosphate efflux, PBMC from the p.N619D patient were significantly impaired in phosphate efflux (Fig. 4b left panel), while phosphate uptake remained unaffected in the patient PBMC (Fig. 4b right panel).

## Discussion

All XPR1 variants demonstrated so far to be causative of PFBC have been mapped within the N-terminal cytoplasmic domain, mostly in the highly conserved SPX domain. Here, we described and characterized for the first time three PFBC-associated variants outside of SPX, one in extracellular loop 3 and two in the C-terminal cytoplasmic region. Moreover, with the *XPR1/SLC53A1* p.(I629S) variant, we report the first mutation that occurred *de novo*. The only non equivocal *de novo* mutations previously identified in PFBC patients were found in PiT2/*SLC20A2*^21^ and *PDGFB*^22^. Moreover, we showed that all three XPR1 variants studied here have impaired phosphate exporter function, while maintaining efficient cell surface expression and retroviral receptor function. Interestingly, a more profound defect in Pi export as tested *in vitro* was consistently observed with the two variants located in the cytoplasmic region.

The three patients reported here showed lenticular and cerebellar hemisphere calcifications that appeared to be less severe than those observed in patients with XPR1 changes located in the SPX-harboring N-terminal cytoplasmic region. However, the number of patients remains too low to perform subgroup analyses that would take into account age as a covariable. Nevertheless, our present characterization and assessment of these three *XPR1* variants, in exons 11 and 14, allowed the reclassification of c.1375C > T p.(R459C) and c.1886T > G p.(I629S) as pathogenic variants (class 5), with an impaired phosphate exporter function likely to play a direct role in pathogenicity. More importantly, our evaluation of variant c.1855A > G p.(N619D), which has been considered so far as of unknown significance, allows now its classification as likely pathogenic (class 4), and therefore its use for genetic counseling.

Depletion of *Xpr1* in mice is lethal, emphasizing the key role of this gene in phosphate homeostasis^23^ and we have previously shown that mutations in the cytoplasmic N-terminal domain of XPR1 associated to PFBC lead to a defective phosphate export *in vitro* and *ex vivo*^6,11^. Also, a *Pit2/Slc20a2* conditional knockout mouse model led to dysregulation of phosphate levels in cerebrospinal fluid and to brain vascular calcifications^24–26^. Although calcium phosphate depositions in PFBC appeared to be due to an alteration of phosphate metabolism, the mechanisms underlying the respective roles of the five genes identified as causative for PFBC remain unsolved. Interestingly, heterozygous variants in *PiT2/SLC20A2*, *PDGFRB*, *PDGFB*, and *XPR1/SLC53A1* are associated with disease development, while only bi-allelic variants in *MYORG* have been described in PFBC^7^.

The SPX domain has been shown to bind inositol pyrophosphate molecules with high affinity^12^. Whether XPR1 C-terminal region also interacts with cytosolic partners or with SPX remains to be studied. Interestingly, XPR1 C-terminal region includes a conserved segment from residues 600 to 635 (numbering is using human XPR1 as a reference), with the cytoplasmic domain predicted to start at L612 (Figs 1d and S2). Conserved region 600–635 is predicted to be mainly in helical conformation (PSIPRED^27^, DISOPRED^28^, MEMSAT^29^), while the very C-terminus sequence, downstream of residue M646, is highly variable and predicted to be natively unfolded (not shown). Isoleucine residue at position 629 is strictly conserved in metazoan (Fig. 1e) and often substituted in other species to the similar and hydrophobic valine (Fig. S2). Conservation of a hydrophobic residue at this position supports that a substitution toward a polar and flexible residue, such as seen with the PFBC p.I629S variant, is damaging. With regard to the PFBC p.N619D variant, it is remarkable that no substitution of N619 toward D619 was observed across species, although Asp residue is the Asn most similar and isosteric residue, mainly differing by a negative charge harbored by Asp. It is therefore likely that the additional charge brought by Asp in PFBC p.N619D prevents proper local folding or interaction with other domains of the protein core or (a) cytoplasmic partner(s). As tested *ex vivo* on the proband PBMC, the p.N619D variant led to a defect of phosphate export in the proband’s cells (Fig. 4b).

Interestingly, the p.(L612_T696del) construct in which the C-terminal cytoplasmic domain has been deleted can be expressed, although it did not appear to reach the cell surface. A similar role of the C-terminal cytoplasmic domain in the plasma membrane trafficking of GLUT1/SLC2A1 has been observed^30^; unpublished observations. On the other hand, this C-terminal cytoplasmic domain also harbors multiple predicted phosphorylation and ubiquitin consensus sites between positions 660 and 690 that may explain trafficking defect. Our data however suggest that partner interaction of the XPR1 C-terminal domain plays a role beyond vesicular trafficking. Therefore, the search for XPR1/SLC53A1 C-terminal interacting partners is likely to further our understanding of the role of this region in phosphate homeostasis and calcification diseases.

The three XPR1 C-terminal variants analyzed here were efficiently expressed, despite that XPR1 p.N619D showed lower level expression. Moreover, when introduced into an XPR1 expression vector, the three mutations did not alter the capability of XPR1 to serve as retroviral receptor for X-MLV. Therefore, the XPR1 variants reported here seem to exclusively alter phosphate export, although at different levels, with impact neither on expression of the phosphate importers PiT1/SLC20A1 and PiT2/SLC20A2, nor on phosphate uptake. In this sense, the XPR1 C-terminal variants are similar to the PFBC variants of the SPX domain^6,11^. *XPR1/SLC53A1* heterozygous variants are not the most common cause of PFBC^2^; however, all the functional analyses of damaging variants resulted in phosphate efflux impairment. Importantly, this study is the first one to report a *de novo* XPR1 mutation causing PFBC p.(I629S). Moreover, we characterized clinically and functionally for the first time *XPR1*/*SLC53A1* variants located outside of the region that encodes the SPX-harboring N-terminal cytoplasmic domain, unveiling a specific role of the XPR1 C-terminal domain in phosphate transport and PFBC. The nature and evolutionary residue conservation of the two variants of the C-terminal domain, p.N619D and p.I629S (Figs 1e and S2), as well as their specific impact on phosphate export, suggested that the former mutation likely impacted on a direct protein interaction, either with cellular partner(s) or intramolecularly, whereas the latter more likely leads to changes in a local structural conformation.

## Patients, Materials and Methods

### Guidelines and regulations

All patients or legal guardians, and unaffected relatives when involved, provided informed written consent for genetic analyses and experimental protocols that were approved by the *Comité de Protection des Personnes (CPP) Ile de France II* ethics committee. All experiments with human and animal samples and cell lines were performed in accordance with guidelines and regulations set by the French *Centre National de la Recherche Scientifique* (CNRS), and the *Institut National de la Santé et la Recherche Médicale* (INSERM).

### Patients

Patients were recruited by the French PFBC study group from multiple French centers as previously described^31^. Medical charts and DNA blood samples were sent to Inserm U1245, Rouen, France, for genetic analyses, with the aforementioned ethics committee approval. *XPR1* screening was performed by Sanger sequencing in patients formerly negatively screened for *SLC20A2* and *PDGFB* variants. Variants were interpreted following the ACMG-AMP recommendations^32^.

After the identification of the I629S variant in patient ROU 5075, which was absent from the parental sample, we confirmed parenthood using a set of four informative microsatellites.

CT scans were analyzed using a previously described method that allowed the rating and computing of patient calcifications as a total calcification score (TCS) on a scale of 0–80^31^.

### Cells

Human HEK293T and Chinese hamster ovary (CHO) cells were cultured in DMEM supplemented with 10% fetal bovine serum (FBS) (PAN-Biotech) and non-essential amino acids. Cells were incubated at 37 °C in a 5% CO2 and humid atmosphere. For phosphate-free experiments, cells were cultured in phosphate-free DMEM supplemented with 10% dialyzed FBS, as previously described^6,10,11^.

### Plasmids and siRNAs

The p.R459C, p.N619D and p.I629S mutations were generated by site-direct mutagenesis using recombinant overlapping PCR and introduced in the pCHIX expression vector, which contains two copies of the HA tag^33^. HA-tagged versions of the *XPR1* variants were also inserted into a pLNCX retroviral vector^34^. Small interfering RNAs (siRNA) (Integrated DNA Technologies) targeting the 3′ UTR of human *XPR1/SLC53A1* were as follows: 5′-ugauauaacuccugugcaatt-3′ and 5′-ccuuaacagcugaagcuautt-3′. siRNA directed against the firefly *luciferase* gene was used as control. HEK293T cells grown on poly-D-lysine-coated 6-well plates were transfected with 50 pmol siRNA per well, using the calcium phosphate method along with either empty or *XPR1/SLC53A1*-harboring expression vectors.

### Phosphate fluxes

Phosphate uptake and efflux assays in HEK293T cells were carried out as previously described^10^. Amount of phosphate uptake was calculated from the concentration of cold phosphate in the medium multiplied by the ratio of cellular [^33^P]phosphate to total [^33^P]phosphate supplemented within a period of 30 minutes. Percentage of phosphate efflux was calculated as the ratio of released [^33^P]phosphate to total cellular [^33^P]phosphate. Phosphate uptake and efflux assays in peripheral blood mononuclear cells (PBMC) were performed as previously described^6,11^.

### Immunoblotting

Whole cell extracts were separated by 12% SDS-PAGE under reducing conditions and transferred to PVDF membranes. Proteins were detected with antibodies against HA (3F10, Roche; 1:5000) or β-actin (A5441, Sigma Aldrich; 1:5000). Proteins of interest were detected with horseradish peroxidase (HRP)-conjugated anti-mouse or anti-rat antibodies (Southern Biotech; 1:5000). Visualization was performed with Pierce ECL western blotting substrate (Thermo Scientific).

### Flow cytometry

Cell surface expression of phosphate transporters was monitored on HEK293T cells with soluble ligands derived from the receptor-binding domain (RBD) of different retroviral Envs. RBD derived from mouse X-MLV, koala endogenous retrovirus, and mouse amphotropic-MLV Envs, were used to detect XPR1/SLC53A1, PiT1/SLC20A1 and PiT2/SLC20A2, respectively, and binding assays performed as previously described^10,35,36^. Control expression of the GLUT1 glucose transporter was monitored with the GLUT1-specific H2RBD ligand, derived from the human T-cell leukemia virus type 2 Env^30,33^. Briefly, 5 × 10^5^ cells were incubated in PBA (PBS complemented with 2% FBS) containing the adequate RBD, for 30 min at 37 °C under agitation, followed by two washes with PBA and incubation with an Alexa Fluor 488-conjugated anti-mouse IgG1 antibody (Life Technologies; 1:5000) for 20 min at 4 °C. Cells were promptly analyzed on NovoCyte flow cytometer (Becton Dickinson), and data were analyzed with the FlowJo package. All RBD ligands were produced as previously described^10,35^, or obtained from METAFORA-biosystems.

### Virus production

EGFP virus vectors were produced in 2 × 10^6^ HEK293T cells in 10 cm dishes, co-transfected using calcium phosphate with a combination of 10 µg of the MLV-based LNCG retroviral vector carrying the EGFP reporter gene^37^, with 5 µg of the MLV Gag/Pol expression vector (pC57GPBEB)^38^, and 5 µg of either the vesicular stomatitis virus (VSV) G protein, or the X-MLV Env expression vectors. Virion-containing media were harvested 2 days later, filtered through 0.45 µm (pore size) filter and stored at -80 °C before use. LNCX virus vectors carrying *WT* and mutated *XPR1/SLC53A1* genes were produced in the same conditions, except that the LNCG retroviral vector was replaced by the various *XPR1/SLC53A1* LNCX vectors.

### Selection of cells stably producing XPR1 variants, and retrovirus infection

CHO cells stably expressing XPR1 constructs were generated by transducing CHO cells with the pLNC(XPR1), pLNC(XPR1R459C), pLNC(XPR1N619D) or pLNC(XPR1I629S) vectors, or the empty pLNCX vector, and selected the next day with medium containing 1.5 mg per ml of G418 (active fraction). G418-resistant clones were pooled after 2 weeks of selection before further experiments.

CHO cells that stably expressed WT, p.R459C, p.N619D or p.I629S XPR1/SLC53A1 were plated in 12-well plates with 2 × 10^4^ cells per well. Infections were performed the following day with serial dilutions of replication-defective LNCG retroviral vector pseudotyped with either X-MLV Env or VSV-G glycoproteins. Percentages of EGFP-positive cells were analyzed by flow cytometry.

### Statistical analysis

The Student’s *t* test was applied using GraphPad Prism 6 software; P values were as follows: *P ≤ 0.05; **P ≤ 0.01; ***P ≤ 0.001; ****P ≤ 0.0001.

## Supplementary information

Figures_Suppl 1–2.pdf
