# Supplementary material for: Characterization of XPR1/SLC53A1 variants located outside of the SPX domain in patients with primary familial brain calcification
Source: Sci Rep. 2019 May 1;9:6776. doi: 10.1038/s41598-019-43255-x (PMC6494797; doi:10.1038/s41598-019-43255-x)
Supplement: Supplementary file 1 — Figures_Suppl 1–2.pdf [file 41598_2019_43255_MOESM1_ESM.pdf]

SUPPLEMENTAL FIGURES

Characterization of XPR1/SLC53A1 variants located outside of the SPX domain in patients with primary familial brain calcification

Uriel López-Sánchez[a], Gaël Nicolas[b], Anne-Claire Richard[b], David Maltête[c], Mahmoud Charif[d], Xavier Ayrignac[d], Cyril Goizet[e], Jawida Touhami[a], Gilles Labesse[f], Jean-Luc Battini*[a,g] and Marc Sitbon*[a]

[a] Institut de Génétique Moléculaire de Montpellier, University of Montpellier, CNRS, Montpellier, France

[b] Normandie Univ, UNIROUEN; Inserm U1245; Rouen University Hospital, Department of Genetics and CNR-MAJ; Normandy Center for Genomic and Personalized Medicine, Rouen, France

[c] Department of Neurology, Rouen University Hospital and University of Rouen, France; INSERM U1239, Laboratory of Neuronal and Neuroendocrine Differentiation and Communication, Mont-Saint-Aignan, France

[d] Department of Neurology, Montpellier University Hospital, Montpellier, France

[e] INSERM U1211, Univ Bordeaux, Laboratoire Maladies Rares, Génétique et Métabolisme; CHU Bordeaux, Service de Génétique Médicale, Bordeaux, France

[f] Centre de Biochimie Structurale, University of Montpellier, CNRS, Montpellier, France

[g] Institut de Recherche en lnfectiologie de Montpellier, University of Montpellier, CNRS, Montpellier, France

* Corresponding authors :

Correspondence to jean-luc.battini@irim.cnrs.fr or marc.sitbon@igmm.cnrs.fr

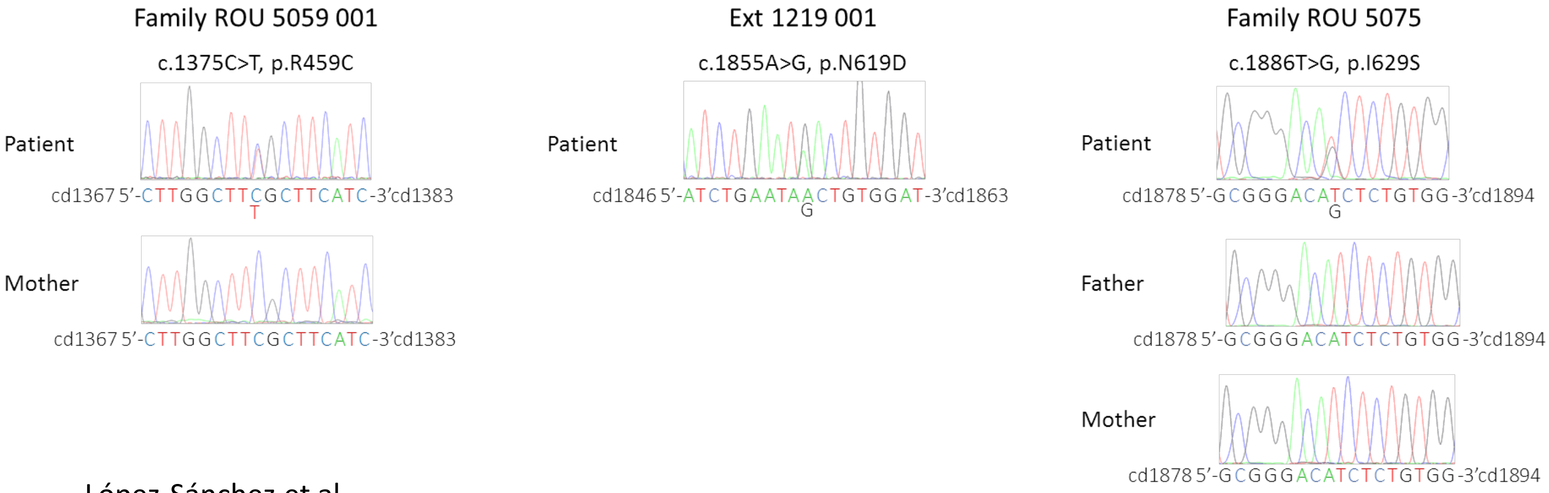

López-Sánchez et al.

Supplemental Figure S1.

Figure S1. Identification of XPR1/SLC53A1 variants in families with PFBC patients. DNA sequence chromatograms showing the different heterozygous variants in XPR1/SLC53A1 evaluated in this study. Available DNA sequences from family members without PFBC are also shown. Variants c.1375C>T p.(R459C) and c.1855A>G p.(N619D) have been recently recorded (ref. 13 of the manuscript). Chromatograms of ROU 5075 patient unveil the de novo mutation c.1886T>G p.(I629S) identified in this study.

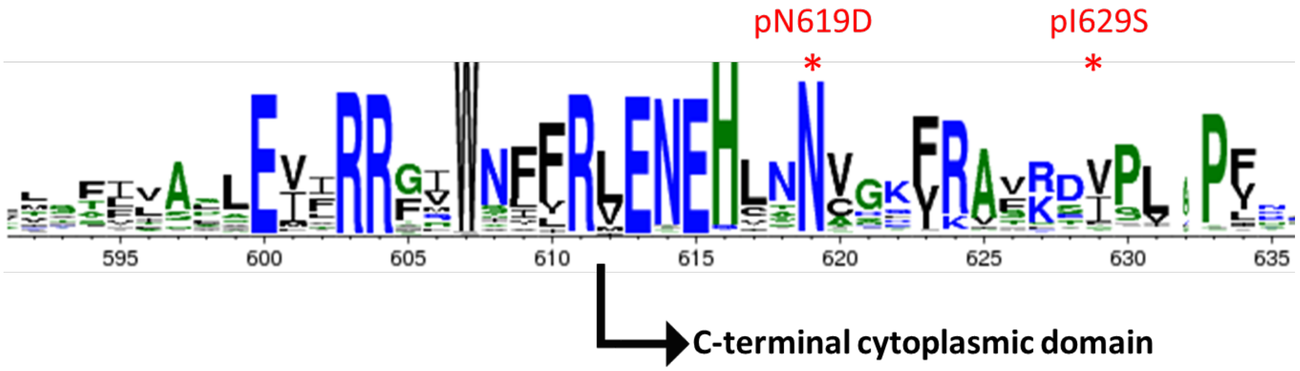

López-Sánchez et al.

Supplemental Figure S2.

Figure S2. Evolutionary conservation of XPR1/SLC53A1 residues in the C-terminal cytoplasmic domain harboring the PFBC variants. Residue conservation in the C-terminal region of XPR1/SLC53A1 across metazoan and protozan orthologs. N619D and I629S PFBC variants are indicated, as well as the predicted C-terminal cytoplasmic domain (MEMSAT-SVM modelisation). Residue numbering is according human XPR1.
